# Mixed-mechanism analgesics for mixed pain

**DOI:** 10.3389/fmed.2025.1717207

**Published:** 2025-11-26

**Authors:** Robert B. Raffa

**Affiliations:** Temple University, Philadelphia, PA, United States

**Keywords:** mixed pain, analgesics, mechanism of action, ascending pathways, descending pathways, synergy

## Abstract

The concept of “mixed pain” simultaneously highlights the benefit of integration of emerging advances in basic science (in particular, physiologic mechanisms) with advances in clinical care (symptomatology and diagnosis), and highlights some current prevailing challenges for both. This dichotomy is reflected in the very definitions or descriptions of mixed pain as a type of pain that manifests with symptoms that include those indicative of both nociceptive pain (viz., pain arising from tissue damage) and of neuropathic pain (i.e., pain arising from nerve damage), and possibly including other types of pain. The dichotomy also underscores the difficulty that is encountered treating such condition(s). Pharmacologic approaches are often accompanied by a variety of complementary non-pharmacologic approaches such as physical therapy, cognitive-behavioral therapy, immune and nutritional boosters, and a host of other modalities aimed at the attenuation of pain. This Perspective reviews the centrally-acting analgesics that have mixed mechanisms of analgesic action.

## Introduction

If pain is best treated by matching the underlying pain mechanism(s) with an analgesic that has a correspondingly-directed mechanism of action (1), and mixed pain is comprised of a combination of pain types (2–4), interventions having a combination of mechanistic approaches (pharmacologic as well as non-pharmacologic) might be more effective than an approach that involves only a single mechanism of action (5). By extension, pharmacotherapy that engages more than one mechanism of action would seem to be a desirable approach. Ideal would be a drug that is effective against nociceptive, neuropathic, nociplastic, inflammatory, and any other kind of pain. But no such analgesic magic bullet exists. Therefore, such broad coverage can only be approximated by combination therapy – either by using multiple single-mechanism drugs, or by using drugs with multi-mechanisms of analgesic action. An additional advantage of using a combination approach is the potential for achievement of additive or even synergistic interaction between/among the mechanisms of action (6). This perspective focuses on single-agent centrally-acting multi-mechanistic analgesics.

## Multi-mechanistic analgesics

**Figure 1:**
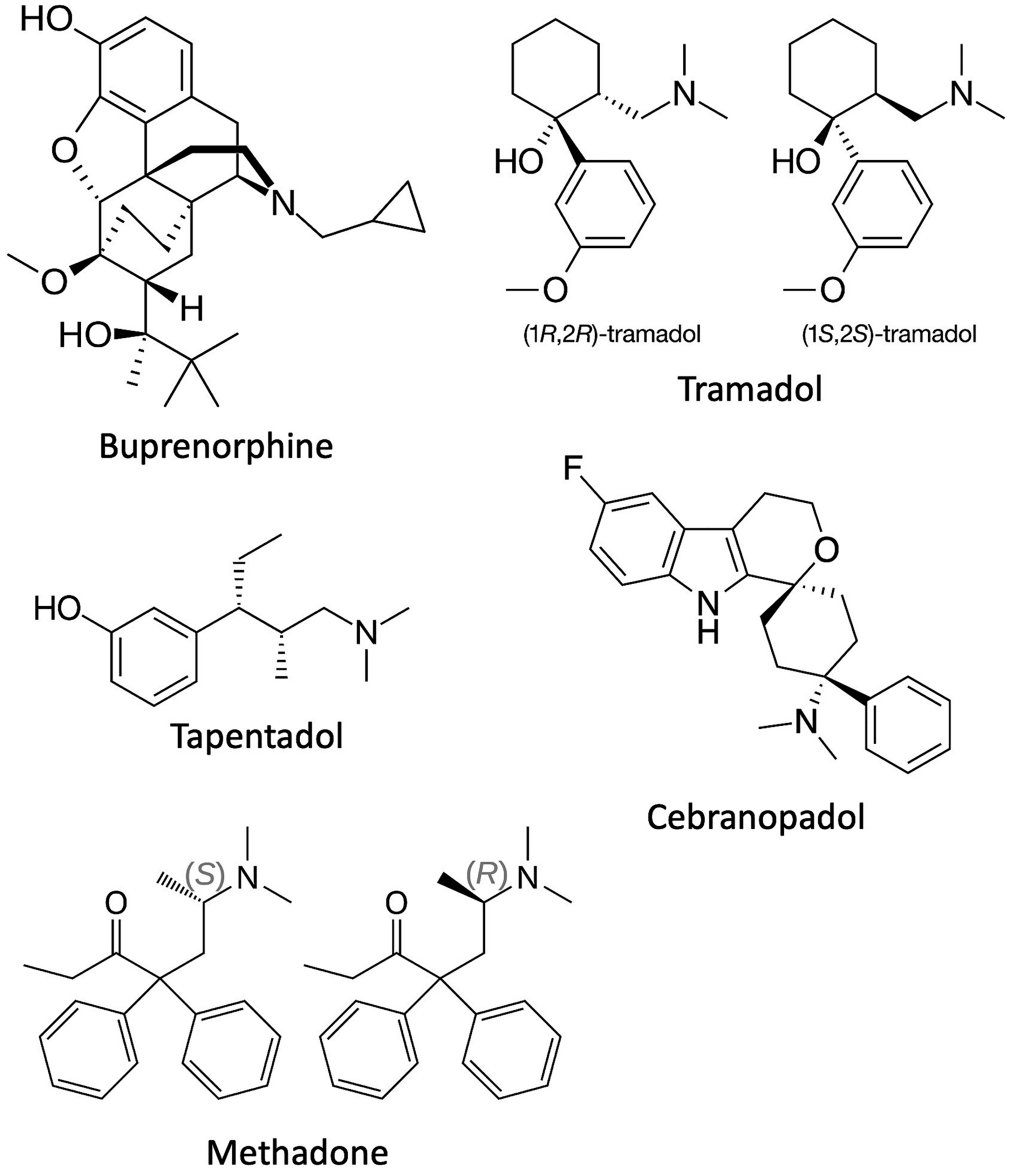
The chemical structures of the centrally-acting multi-mechanistic analgesics reviewed in this perspective.

There are probably several analgesics that might have multi-mechanistic contributions to their overall clinical analgesic effects. However, only a few have been well-documented, and the clinically-relevant contributions of the component mechanisms demonstrated in animal, and in some cases human, studies. By chronological appearance, these drugs are buprenorphine, tramadol, tapentadol, and cebranopadol, with methadone discussed separately (Figure 1).

**Figure 2:**
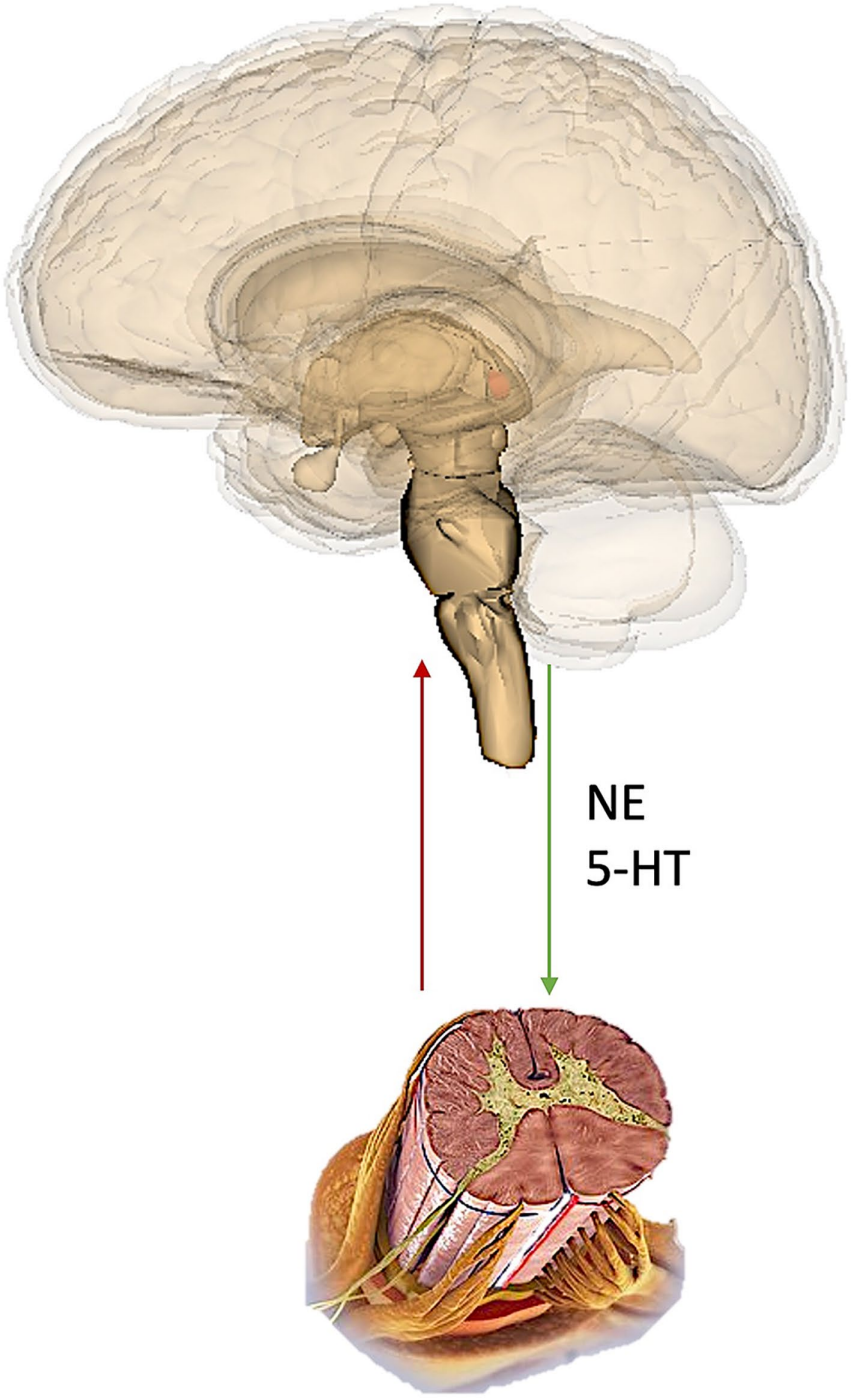
Representation of the ascending and descending pathways. NE, norepinephrine; 5-HT, serotonin (5-Hydroxytryptamine).

A commonality of these analgesics is that they in some way interact with both “ascending” pain signal-transmitting pathways, and also “descending” signal-modulating pathways (Figure 2).

### Buprenorphine

Buprenorphine was first synthesized in the mid 1960’s and has antinociceptive and analgesic activity against a wide variety of pains, including nociceptive, musculoskeletal, neuropathic and cancer-related pains (7, 8). Buprenorphine’s oral absorption is limited, but its physiochemical properties make it suited for administration by other routes.

Buprenorphine has high affinity for MOR (low nM range), but low *in vitro* intrinsic-activity as measured by [^35^S]GTPγS binding in several receptor binding assays (9). The latter led to characterization of buprenorphine as a “partial” agonist, because it produces <100% 2^nd^-messenger activation than that produced by some comparator “full” agonists. However, in the same *in vitro* assays in which buprenorphine produces <100% effect, morphine does also (a fact perhaps not widely known) (10).

Buprenorphine has a multifaceted pharmacology, such that its total analgesic effect derives from activity involving several analgesic mechanisms (11), perhaps even including nonopioid (12). The exact contribution of each of these mechanisms alone or in combination is not fully known.

The basis for the classification of buprenorphine as a “partial agonist” has led to some confusion. This is because when a drug is characterized as a “full” or “partial” agonist based primarily on *in vitro* assays (as was the case for buprenorphine), it can confuse the related but distinct, pharmacologic principles of “affinity” and “intrinsic-activity”, which are properties at the receptor level, with “efficacy”, which is manifested at a particular endpoint. “Affinity” is the thermodynamically-driven chemical attraction between a drug and a receptor (13), and “intrinsic-activity” is the biological stimulus imparted by a drug to its receptor (14); whereas “efficacy” indicates the level of drug-induced effect in a given application. Unfortunately, the term “efficacy” is often loosely used as if it is a fundamental property of a drug, rather than as being situation (endpoint) dependent. A term such as “clinical efficacy” would be clearer. This distinction has led, for example, to confusion in advice about predicted concerns about switching from a “full” agonist analgesic to buprenorphine. Due to differences in 2^nd^-messenger coupling, biased agonism, or other signal transduction phenomena, a drug can act as a full agonist on one endpoint (e.g., analgesia) and as a partial agonist on another (e.g., respiratory depression). Because buprenorphine displays greater than 98% antinociceptive efficacy in the majority of animal models, and positron emission tomography (PET) scans of human brain suggest that less than 100% of MOR are occupied at analgesic doses,14 there only remains the relevant clinical question: does buprenorphine produce equivalent analgesia to a drug that is considered to be a full agonist? That is, does buprenorphine act as a full agonist in clinical pain settings? In a review that examined this question (15), 24 controlled clinical trials were identified, plus a case report and a dose–response curve. Based on complete or comparable pain relief, buprenorphine displayed full clinical analgesic efficacy in 25 of the 26 studies.

### Tramadol

Tramadol originated from a discovery program that was designed to identify novel opioid analgesics. The complete picture of its multi mechanisms of action was elucidated following recognition of, and in an effort to explain, its better clinical profile compared to traditional opioids. It is now known from a large number of studies that its analgesic mechanisms do not reside in a single molecule, but instead in a fortuitously balanced combination of parent and metabolite molecule and the enantiomers of each (when used at doses that take advantage of the contribution of the combination) (16).

Racemic tramadol has quite low affinity for *μ*-opioid receptors (MOR), namely, about 1/10^th^ that of codeine (17–19). The (+)-enantiomer has affinity equivalent to that of dextromethorphan. The M1 (*O*-desmethyl) metabolite of tramadol has higher affinity for MOR than does the parent drug (about 1/10^th^ that of morphine), and an opioid component contributes to tramadol-induced antinociception in animals and analgesia in humans. However, because the opioid antagonist naloxone does not reverse all of the antinociceptive or analgesic effects of tramadol in animals or humans (18, 20), additional naloxone-resistant, i.e., non-opioid, mechanisms must contribute significantly to its antinociceptive and analgesic effects (so it is not a prodrug when used at appropriate doses). The search for the non-opioid mechanisms of action revealed that tramadol inhibits the neuronal re-uptake of norepinephrine and serotonin (21) by binding to the norepinephrine and serotonin reuptake transporters (NET and SERT, respectively) with about the same affinity as it binds with MOR, and at appropriate doses, about equally contributes to the overall analgesic action. Synergistic interaction between the two enantiomers has been demonstrated (19). The combination of mechanisms of action also appear to explain the reduced incidences of respiratory depression and other adverse opioid effects relative to traditional opioids.

Because the opioid, adrenergic, and serotonergic activity of tramadol predominate differentially in the (+) and (−) enantiomers of the parent, and in the (+) and (−) enantiomers of the M1 metabolite, tramadol can be interpreted as a combination of several molecules, each with different pharmacological and pharmacokinetic properties. The fortuitous combination of these molecules leads to beneficial clinical attributes of the drug.

### Tapentadol

In contrast to the serendipitous combination of analgesic mechanisms of tramadol uncovered post-marketing ex-USA, tapentadol was designed to rationally combine two mechanisms of analgesic action, namely, MOR agonism and inhibition of neuronal norepinephrine reuptake inhibition within a single molecule (22).

Based on the favorable clinical experience with the multimodal analgesic mechanism of action of tramadol, a research program was initiated to design other analgesics that would combine MOR agonism and norepinephrine reuptake inhibition, but with minimal serotonergic activity (23). In order to simplify the pharmacokinetic aspects of tramadol, it was also desired that both mechanisms of the new compounds would reside in a single molecule. The research program led to tapentadol. The pronounced 3D differences in chemical structure of tramadol and tapentadol translate into a number of functional differences between the two. Tapentadol binds to recombinantly expressed human MOR (hMOR) with an affinity (K_i_ value) of 0.16 μM (21). Thus, tapentadol has a higher affinity for hMOR than does tramadol, but still orders of magnitude less than morphine. Tapentadol binds to human hNET and hSERT with K_i_ values of 8.8 and 5.28 μM (21). Thus, tapentadol is slightly more potent at inhibiting hNET than is tramadol, but it is nearly five-fold less potent than is tramadol in inhibiting rSERT.

That the two mechanisms of action of tapentadol show a synergistic interaction with respect to antinociceptive activity was demonstrated in a tail-flick test in the rat (24). Indirect evidence was also obtained in that study for an intrinsic antihypersensitive synergy in a neuropathic pain model. To the author’s knowledge, this was the first demonstration of two mechanisms of action (MOR agonism and NRI) interacting to produce a synergistic effect within one molecule.

A practical positive attribute of tapentadol is that it is mainly metabolized by glucuronidation, which avoids an interaction with the larger number of drugs that are metabolized via the CYP system.

### Cebranopadol

Cebranopadol is a novel multi-mechanistic single-molecule analgesic that is currently in clinical development. Its analgesic action appears to be primarily attributable to balanced agonist action at MOR and NOP (nociception/orphanin FQ) receptors (25, 26), with perhaps the contribution of other receptors (27, 28). Although buprenorphine also has MOR and NOP activity, cebranopadol, unlike buprenorphine, has essentially the same affinity at the two sites. NOP in humans is a G protein-coupled receptor that was originally called “opioid receptor-like-1 (ORL-1)” receptor, because it has a high sequence homology to opioid receptors. However, it does not interact with opioid drugs (29), hence the name ORL-1 seemed inappropriate. When the endogenous peptide ligand for this receptor was identified as “nociceptin/orphanin FQ” (N/OFQ), the receptor was renamed NOP. Agonist binding to NOP reduces afferent neuronal excitability and neurotransmitter release by inhibiting cAMP formation, closing voltage-gated Ca^2+^ channels, and opening inwardly-rectifying K^+^ channels, all of which contribute to an analgesic effect (30).

Cebranopadol displays highest affinity and intrinsic activity at MOR (K_i_ = 0.7 nM, 100%) and NOP (K_i_ = 0.9 nM, 89%). Affinity or intrinsic-activity is lower at the other opioid receptor subtypes, and affinity at more than 100 other (off-target) receptors, ion channels, and enzymes is 100- to 1,000-fold lower than at MOR and NOP. *In vivo*, cebranopadol displays antinociceptive, antihyperalgesic, and anti-allodynic properties in neuropathic pain models (31). Cebranopadol is significantly more potent than morphine in these tests and it is relatively more potent in animal models of chronic than it is in models of acute pain.

Thus, cebranopadol – by balanced combined agonist action at MOR and NOP – provides highly potent and efficacious antinociception in various pain models with a favorable side-effect profile. The precise nature and extent of cebranopadol’s clinical profile and utility will depend on the experience in clinical use.

### Methadone

Methadone was discovered in Germany in the latter half of the 1930s, then was named and marketed about 10 years later. Methadone is said to have more than one mechanism of analgesic action: one being opioid and the other(s) being non-opioid, often stated to be related to the NMDA (*N*-Methyl-D-aspartate) subtype of glutamate receptor (32). However, the evidence is not clear cut.

Methadone is a racemate, (*R,S*)-MTD, and its enantiomers display differing pharmacology (33). For example, the affinity of (*R,S*)-MTD at rMOR is 16 nM (K_i_ value), the affinity of (*R*)-MTD at the same receptor is 8 nM, and the affinity of (*S*)-MTD is 61 nM. Although the affinity of (*R*)-MTD is comparable to that of hydrocodone and oxycodone, the fact that the affinity of (*R*)-MTD and (*S*)-MTD are greater and less than that of (*R,S*)-MTD, respectively, indicates that the presence of (*S*)-MTD reduces the affinity of the racemic mixture (*R,S*)-MTD (i.e., methadone). Furthermore, 2^nd^-messenger activation studies (agonist-stimulated [^35^S]GTPγS) show that (*S*)-MTD is only a partial agonist at MOR. These receptor-level differences are manifested as differences in *in vivo* antinociceptive activity: the antinociceptive activity (increase in hot-plate latency) dose–response curve of (*R,S*)-MTD is displaced to the right of (*R*)-MTD due to the approximate two orders of magnitude rightward shift of (*S*)-MTD compared to (*S*)-MTD. Thus, the presence of (*S*)-MTD reduces the antinociceptive activity of the racemic mixture (*R,S*)-MTD (methadone).

Regarding the putative activity of methadone at the NMDA receptor, the binding affinity of (*R,S*)-MTD at the rNMDAR is 1,960 nM, which is about 100-fold less than its affinity at rMOR (33). The affinity of (*R*)-MTD and (*S*)-MTD are similar to that of (*R,S*)-MTD. Displacement of [^3^H]MK801 (an NMDAR antagonist) in brain scans mirror the low affinity. Thus, there is not strong evidence for a demonstrable additional mechanism of analgesic action of methadone (20).

## Discussion

Mixed pain is comprised of a combination of pain types. If pain is best treated by matching the pain mechanism(s) with a correspondingly-directed analgesic mechanism of action, analgesics having a combination of mechanistic actions should be more effective than an approach that involves only a single mechanism of action. Therefore, pharmacotherapy that engages more than one mechanism of action would seem to be a desirable approach for mixed pain. Ideal would be a drug that is effective against nociceptive, neuropathic, nociplastic, inflammatory, and any other kind of pain. But no such single analgesic currently exists. Thus, such broad coverage can only be approximated by combination therapy – either by using multiple single-mechanism drugs, or by using drugs with multi-mechanisms of analgesic action. An additional advantage of using a combination approach is the potential for additive or even synergistic interaction between/among the mechanisms of action.

Buprenorphine combines very high affinity for MOR with several additional known analgesic mechanisms, including even possibly, non-opioid mechanisms.

Tramadol combines the fortuitous pharmacologic activity of parent drug with its M1 metabolite, and the enantiomers of each. Each of these molecules have differing pharmacologic profiles, but to differing degrees includes weak (parent) or high (M1) affinity with MOR and inhibition of the neuronal reuptake of norepinephrine and serotonin. The enantiomers demonstrate synergistic analgesic effect, but not adverse-effect synergy.

Tapentadol, built upon the beneficial characteristics of tramadol (Raff et vs.) was designed *de novo* to be a single molecule without active metabolite, and to combine affinity for MOR and inhibition of neuronal reuptake of norepinephrine. The synergistic interaction between the two mechanisms results in high potency analgesic effect, with higher potency in models of neuropathic pain than nociceptive pain. Metabolism mainly by glucuronidation reduces the chances of a drug–drug interaction.

Cebranopadol combines agonist action at MOR and NOP with about the same binding affinity and 2^nd^-messenger intrinsic-activity. It demonstrates good efficacy and safety in a variety of preclinical models of acute pain, and has particularly potent efficacy in preclinical models of neuropathic pain. The adverse-effect profile of cebranopadol in these tests is reported to be superior to morphine at equianalgesic doses. If it receives regulatory approval, cebranopadol would represent the first truly novel centrally acting muti-mechanistic analgesic in several years.

Methadone, which is a racemate that consists of enantiomers that have differing and in some cases opposing pharmacology, is clearly a MOR agonist, but a putative second mechanism of analgesic action is difficult to substantiate.

Based on the current understanding of mixed pain, namely that it is a composite of pains that are multi-mechanistic in their (patho)physiology, analgesics that have multi-mechanistic mechanisms of action should be more effective than single-mechanism analgesics.

## Data Availability

The original contributions presented in the study are included in the article/supplementary material, further inquiries can be directed to the corresponding author.
